# Chiral resolution of spin angular momentum in linearly polarized and unpolarized light

**DOI:** 10.1038/srep16926

**Published:** 2015-11-20

**Authors:** R. J. Hernández, A. Mazzulla, C. Provenzano, P. Pagliusi, G. Cipparrone

**Affiliations:** 1 Physics Department, University of Calabria, Ponte P. Bucci, Cubo 33B, 87036 Rende (CS), Italy; 2 CNR-Nanotec, LiCryL lab., Ponte P. Bucci, Cubo 33B, 87036 Rende (CS), Italy

## Abstract

Linearly polarized (LP) and unpolarized (UP) light are racemic entities since they can be described as superposition of opposite circularly polarized (CP) components of equal amplitude. As a consequence they do not carry spin angular momentum. Chiral resolution of a racemate, i.e. separation of their chiral components, is usually performed via asymmetric interaction with a chiral entity. In this paper we provide an experimental evidence of the chiral resolution of linearly polarized and unpolarized Gaussian beams through the transfer of spin angular momentum to chiral microparticles. Due to the interplay between linear and angular momentum exchange, basic manipulation tasks, as trapping, spinning or orbiting of micro-objects, can be performed by light with zero helicity. The results might broaden the perspectives for development of miniaturized and cost-effective devices.

Photons have both linear and angular momentum and the effects of one or both become evident in their interaction with matter when scattering or absorption processes occur. The transfer of linear momentum from light to micro-objects is at the basis of the optical tweezers operation principle, where the optical forces are used for trapping and cooling^1^,^2^,^3^. In particular, the transfer of angular momentum from light to matter occurs in two independent forms: 1) spin angular momentum (SAM) which is associated with the light polarization helicity, resulting in the rotation of the particle around its own axis, 2) orbital angular momentum (OAM) which arises from the spatial distribution of the intensity and phase front of the light beam, producing a rotation of the particle around the beam axis^4^,^5^,^6^,^7^,^8^,^9^,^10^. Although in the nonparaxial case the separation of optical angular momentum into its spin and orbital components is more complicated and there are still unsolved questions on this subject^11^,^12^,^13^, the ability to generate optical torques at micro scale from both (SAM or OAM) have been widely employed for mechanical applications like micro-pumps, energy conversion, manipulation^14^,^15^,^16^,^17^,^18^. Accordingly, several control parameters for the optical torque on micro-objects have been investigated and reported in literature^14^,^15^,^16^,^17^,^18^,^19^,^20^,^21^,^22^,^23^,^24^. However, these objects often play a passive role in determining the mechanical effects related to the transfer of angular momentum from the light. On shape-symmetric isotropic particles, only the light that has non-vanishing (spin or orbital) angular momentum is able to exert a torque, whose sign is guided by the chirality of the light. Recently, the interaction of light with micro-objects based on chiral materials made evidence of several capabilities in optical manipulation originated from the coupling of transferred linear and angular momentum mediated by the chirality of the materials^25^,^26^,^27^,^28^,^29^,^30^,^31^,^32^.

Spherulitic microparticles obtained from photo-polymerized cholesteric liquid crystal (CLC) droplets^33^, owing to their supramolecular helical structures, exhibit selective Bragg reflection phenomenon for light propagating along the helical axis and wavelength within the stop-band. Under these conditions they behave as omnidirectional chiral mirrors^25^,^27^,^33^, i.e. the light with the same handedness of the material is selectively reflected, while the light outside the stop-band or with opposite handedness is completely transmitted. The circularly polarized (CP) light reflected from the chiral mirrors preserves the handedness of the incident one, contrary to what occurs for conventional reflectors that reverse the light helicity. Thus, by conservation of the angular momentum, the chiral particles gain mechanical angular momentum from the CP light with the same handedness and experience a torque. Therefore, they play an active role in the process, allowing transfer of SAM only from proper chiral component of the light, yielding a chiral resolution.

Here we report an investigation of the optical torque exerted by a LP or UP Gaussian laser beam on solid chiral microparticles, namely spherical microparticles with a left-handed molecular spherulitic configuration, immersed in water. We demonstrate the racemic resolution of achiral light through the interaction with chiral matter and a net optical torque on chiral particles depending only on their own handedness. The control of reflectance at the laser wavelength allows to trap and set in rotation the microparticles even in a single beam configuration, thanks to the combined effect of the linear and angular momentum transfer. Stable trap can be achieved either on the beam axis or on an annular region around it, depending on the spot size, the microparticles radius and the reflectance. At the same time, the left-handed CP component of the LP or UP light beam induces spinning or orbital motion of the trapped microparticles. On the basis of this phenomenon, the investigation of the orbital motion and the measurements of optical torque induced by a LP and UP light have been reported. We demonstrate that the rotation sense is solely determined by the particle chirality (i.e. handedness of the supramolecular helical structure), and it is not conditioned by the helicity of light polarization. Remarkably, stable trapping and optical rotation of chiral microobjects, both spinning and orbital motion, can be achieved by a light beam without any predetermined phase distribution^13^ (OAM) or polarization state^7^ (SAM), by properly tuning the light wavelength. In contrast to the actual trend of optical manipulation engineering, that moves towards an increasing level of complexity of geometries, shape or structure of light beams, the reported results open the route for new strategies of opto-mechanical control based on unpolarised and uncoherent light sources, i.e. LED, fulfilling some conditions to develop user friendly devices^34^.

## Results

**Figure 1:**
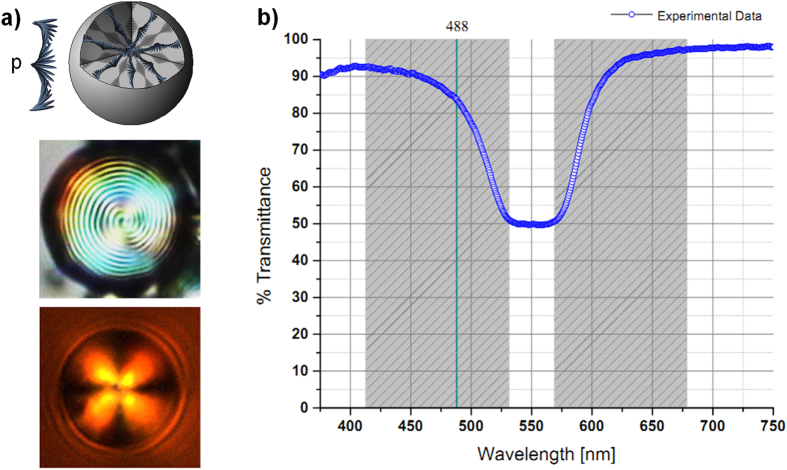
Chiral microparticle and transmission spectrum of a left-handed CLC polymerized film. (**a**) (top) Schematic representation of chiral microparticles with spherulitic configuration, (center) image at the optical microscope between crossed polarizers of microparticles with cholesteric pitch *p* ≅ 1 μm, dark and bright concentric ring circles show the onion-like arrangement of the supramolecular layers of thickness *p/2*, (bottom) image at the optical microscope between crossed polarizers of microparticles with cholesteric pitch *p* ≅ 0.3 μm; for pitch smaller than the light wavelength, the image of the microparticles between crossed polarizers shows the typical Maltese cross [33]; (**b**) Transmission spectrum of a polymerized 15 μm-thick film of left-handed CLC with a chiral dopant concentration of about 23% wt. The gray dashed regions represent the edges of the stop-band *Δλ* and in the present case are about 100 nm large, the solid vertical line indicates the position of the laser wavelength employed for our experiments at λ = 488 nm.

To demonstrate the possibility to use LP or even UP beam to transfer angular momentum from light to matter through a chiral resolution process, we exploit single Gaussian beam optical manipulation of chiral spherical microparticles exhibiting the Bragg selective reflection phenomenon. The microparticles are made of chiral polymeric LC, that is a chiral optically anisotropic dielectric medium in which the average molecular orientation, described by a unit vector ***n*** (director), rotates by 2π around the axis of the supramolecular helical structure over a distance *p*, named cholesteric pitch. Such a chiral ordering may be right- or left-handed. The propagation of a plane wave along the helix axis is forbidden for circularly polarized with helicity parallel to the one of the material structure and wavelength within a spectral range (named stop-band). This is the well-known circular Bragg reflection phenomenon^35^,^36^, and chiral polymeric particles with spherulitic configuration (Fig. 1a) exhibit omni-directional selective reflection working as chiral spherical mirror^33^,^37^. Moreover, polymerized particles with respect to LC droplets prevent deformation or orientation issue of the molecular director during the optical manipulation experiment, establishing unmodified optical properties of the microparticles. To illustrate our concept to combine and control linear and angular momentum transfer in the optical manipulation of chiral objects with achiral light, we use the transmission spectrum of a planar aligned film of CLC for an incident LP light (Fig. 1b). In particular we report the spectrum of a planar 15 μm-thick polymerized film of the reactive mesogen (RMS03-001C by Merck), with a concentration of left-chiral dopant ZLI-811 (23% wt.), providing a cholesteric (left-handed) pitch of 300 nm. The geometrical arrangement of helical axes perpendicular to the glass substrates and the thickness larger than 6*p*, provides a complete photonic band gap structures with a reflectance *R*^+^ (of left CP light) that reaches the 100% value.

Consequently LP light is half-reflected and half-transmitted in the flat spectral region of the stop-band (Fig 1b). The range of the wavelength where the Bragg phenomenon takes place is Δλ* = p*Δ*n*, centred at λ_*B*_=*p*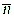, with 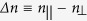 (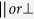
refers to the molecular director), *p* the cholesteric pitch and 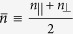 the average refractive index. At the centre of the stop-band, the conditions for the Bragg regime are fulfilled and the (left-handed) Fresnel reflection coefficient can be evaluated by *r*^*+*^= tanh(β*d)*, where 
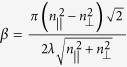
, *d* corresponds to the thickness of material film, λ is the incident light wavelength^35^,^36^. Nevertheless, beyond the flat centre of the stop-band, the total transmittance T changes from 50% to its maximum value, close to 100% because of the negligible absorption. Therefore, the LP light reflectance *R*, along the edges of the band (gray dashed areas in Fig. 1b) can be tuned from 50% to zero, by tuning the light wavelength or the band-gap position.

### Trapping stability of LP Gaussian beam

Investigation on the trapping of chiral particles and droplets reported in the literature have demonstrated that stable traps can be achieved, independently on the light or material chirality, for small enough particles (that means particles with partial band-gap, *R*^+^ < 30%)^27^ or, independently of the particles size (even for *R*^+^ = 100%), using two beams trapping configuration^28^. Moreover, in all cases circularly polarized traps have been considered.

To evaluate the trapping stability of achiral light, i.e. having half left- and half right-circular components, with wavelength within the stop-band edge (*R* = *R*^+^/*2* < *50*%), the optical forces are calculated for a single focused Gaussian beam interacting with the left-handed spherulitic microparticles immersed in water. For this purpose, we adopt the ray-optics approach^25^,^27^ to evaluate the longitudinal scattering (***F***_***s***_) and the transversal gradient (***F***_***g***_) force. The validity of this approach is justified for particle radius *a* much larger than the trapping light wavelength. Indeed, ray optics is generally applicable when the particle size parameter ξ = 2π*a*/λ is much larger than 1. In our experiment, particles with radius larger than 6*p* (*a* > 2 μm and ξ > 25) are investigated. The particle is modelled as a partially reflecting spherical chiral mirror, with a reflectance *R* that ranges from 0 to 0.5 for achiral light, and an average refractive index 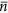.

By considering the usual approach, the recoil optical forces are evaluated from the rate of change of the linear momentum of the light. The power of the light reflected and transmitted are calculated, considering that *R* is dominated by the Bragg phenomenon, whose value varies within the stop-band edge, and *T* = *1-R*.

The force on the sphere can be calculated by [25]:


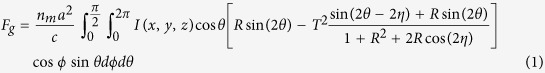



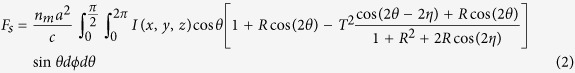


where *n*_*m*_ is the refractive index of the surrounding medium (water), *I*(*x*, *y*, *z*) is the intensity distribution of the Gaussian beam, (*θ*, *ϕ*) are the polar and azimuth angles, respectively in spherical coordinates and *η* is the refraction angle (for details see Supplementary Information).

**Figure 2:**
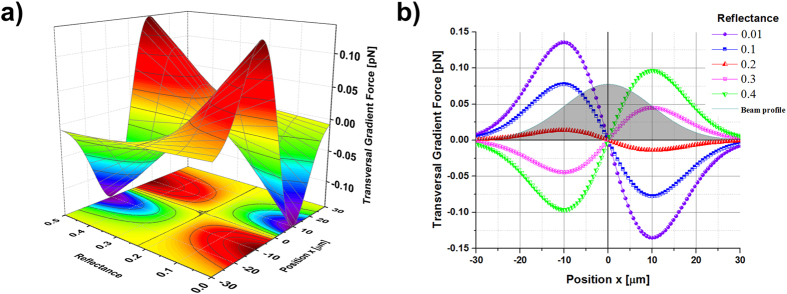
Stability of a LP trap by fixing the particle radius at 2.5 μm. (**a**) Surface plot of the gradient optical force as a function of the particle reflectance. Parameters of the simulation are *w*_*0*_ = 20 μm, *P* = 100 mW, *a* = 2.5 μm. On graphic (**b**) Transverse gradient force versus the transversal position *x* at different reflection values, the gray Gaussian profile represents the light intensity distribution. The reflectance *R* = 0.01 corresponds to a homogenous dielectric particles with a real refractive index 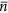.

In Fig. 2 we report our numerical simulation, in which we keep the particle radius *a* = 2.5 μm constant and we assess the stability threshold for a LP trap as a function of the reflectance *R*. This corresponds to moving the stop-band with respect to the light wavelength or vice versa. The graphic a) in Fig. 2 depicts the surface plot of the transverse gradient force ***F***_***g***_ with projection on the plane formed by the reflectance *R* (spanning from 0 to 0.5) against the transversal coordinate *x*, revealing that around the value of *R* = 0.23 there is a narrow threshold that separates two opposite dynamics passing from attractive to repulsive forces. For *R* ≤ 0.23 the net transversal gradient force produces an attractive optical trap with stable equilibrium position located at *x = 0*, corresponding to the light beam intensity peak; while for values with *R* > 0.23 it results in an unstable equilibrium position characterized by a dominant pulling force on the particles that moves them away from the intensity peak. In Fig. 2b), we report the graphs of the transverse optical force ***F***_***g***_ of a LP Gaussian beam calculated for different values of the particle reflectance *R*. The behaviour of the gradient force for *R* ≤ 0.23, is such that the slope of the trapping force calculated at the equilibrium position is in fact always negative. As the reflectance increases, the strength of the trap reduces and an inversion of the slope, which corresponds to an instability condition of the trap, is expected for higher values. Following this scheme, we can completely control the reflectance *R* and the optical trapping by properly tuning the light wavelength with respect to the stop-band or vice versa. In our experiment, the optical trap is expected to be stable, since its wavelength at 488 nm lies within the band edge ensuring a reflectance lower than 0.25 (see Fig. 1).

**Figure 3:**
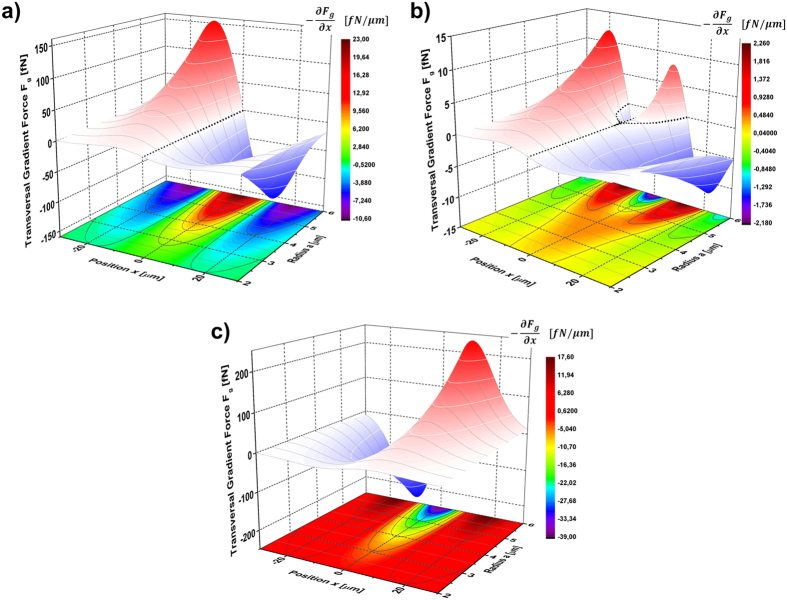
Gradient force and trap stiffness as a function of the radius and the reflectance of the particle. Surface plots of the gradient optical force and flat colour map of the trap stiffness (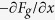) as a function of the particle radius for three different values of the reflectance (**a**) *R = 0.20*, (**b**) *R = 0.22*, (**c**) *R = 0.25*. Parameters of the simulation are *w*_*0*_ = 20 μm, *P* = 100 mW. For *R* = 0.20 stable trapping is always expected at *x* = 0. For *R* = 0.22 stable trapping is achieved at *x* = 0, for particles with a radius lower than 4.4 μm, for larger radius trapping is predicted at *x* ≠ 0, in two symmetric positions with respect to the beam axis. Black dashed lines in (**a,b**) draw the trapping positions of the particles as a function of their radius. For *R* = 0.25 the particles are rejected out of the trap.

**Figure 4:**
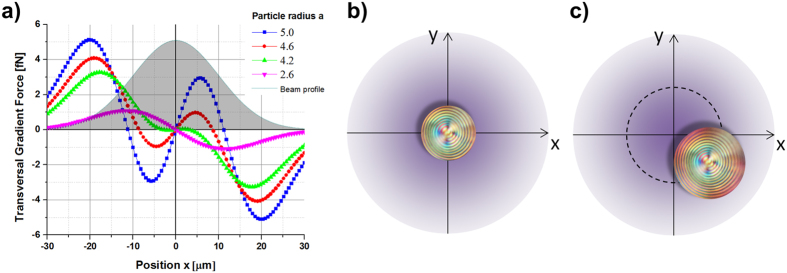
Stable equilibrium positions for particles around the beam axis. (**a**) Transversal gradient force evaluated for different particle radii, for a reflectance *R* = 0.23. The change in the particle size reveals an observable shift in the transverse gradient force equilibrium position away to the peak of intensity giving rise to an annular ring region of stable optical trap. (**b**) Particles with radius smaller than 4 μm are stably trapped in the center of the Gaussian beam on the beam axis. (**c**) Particles with radius larger than 4 μm are stably trapped in an annular region around the beam axis.

It is worth mentioning here that the investigation of the optical force close to the reflectance threshold as a function of the position *x* shows that stable trapping can be also achieved in an annular region around the beam axis. Figure 3 displays the evaluated optical force ***F***_***g***_ and the trap stiffness (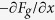) obtained for a Gaussian beam with *w*_*0*_ = 20 μm and reflectance values of 0.20 (a), 0.22 (b), 0.25 (c), for particle radius *a* in the range 2–6 μm. Stable trapping on the beam axis is always expected in the case of R < 0.20. In the range 0.20 < R < 0.25, the trap varies from a single stable equilibrium position centred at the peak intensity (at *x* = 0) for small particle radii, into two symmetric stable equilibrium positions as the radius of the particle increases. Due to the rotational symmetry of the gradient force in the transverse plane (*x*, *y*), those equilibrium positions form an annular ring region of stable optical trapping. The particles are always rejected for R > 0.25. In Fig. 4 we report the graphs of the gradient force *F*_*g*_ at a fixed reflectance value of *R* = 0.23, considering the particle radius ranging from *a* = 2.6 μm to 5.0 μm.

### Spin AM transfer from LP Gaussian beam

Angular momentum is known as an important property of the light after the first theoretical prediction of Poynting in 1909^9^ and the experimental demonstration by Beth in 1936^10^. Moreover, the subsequent studies involving complex and structured light fields have introduced different types of optical angular momentum. Spin AM is associated with the light polarization, so that right- and left-handed circular polarizations of a beam correspond to the positive and negative helicities of the photons^38^. The carried AM of such beam is 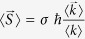 per photon, where *σ* is the helicity parameter, i.e. the degree of circular polarization, and it is aligned with the propagation direction. On the basis of these assumptions, LP light beam does not carry AM.

By investigating the optomechanical effects on chiral microparticles exhibiting the Bragg phenomenon, we have found, from the conservation law of the total angular momentum, that the optical torque of a plane wave in the general case of elliptically polarized light is^27^:


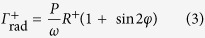


where φ is the ellipticity angle of the light (*φ* = 0 or π/2 corresponds to LP light, 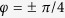 to CP light), *P* is the power of the light beam and ω the angular frequency. Hence we see that 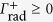, and it vanishes only for the spin-state of light antiparallel to the handedness of the chiral mirror. Thus a radiation torque through transfer of SAM can occur even for light carrying no net spin AM (*i.e*. linear polarization), with a calculated value 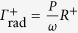. This effect can be accounted for by the broken symmetry in the interaction of the light with the chiral mirror. For a right-handed mirror, the equivalent relation, depending on their own reflectance 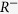, can be found.

It should be noted that, the capability to observe rotational motion of the Bragg particle due to the transfer of AM is conditional on its stable trapping^27^ connected to the linear momentum exchange. Owing to these results, stable trapping can be achieved for LP light whose wavelength ensures reflectance lower than the threshold value between the annular trapping and the rejection regime; a radiation torque 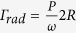 with 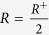 is expected to induce a rotation of the trapped particle.

### Experimental results

A LP Gaussian beam (power *P* = 100 mW and beam waist *w*_0_ = 20 μm) is exploited to demonstrate trapping force and torque on chiral particles with a radius ranging from 2 to 10 μm. Spinning of particles trapped on the beam axis is observed, as well as orbital motion of particles which experience off-axis trapping (see Fig. 3(b)). The rotational frequencies (see Methods) are recovered by direct optical video imaging. A rotational frequency of about 0.5 Hz clockwise (see video 1 in Supplementary Information) is measured for a particle with a radius *a* ≅ 3 μm. The average optical torque Γ_*rad*_ ≈ 2 pN·μm exerted on the particle is evaluated from the balance between the optical and rotational drag torques 
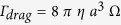
 on a sphere rotating in a fluid at low Reynolds number^8^,^14^.

**Figure 5:**
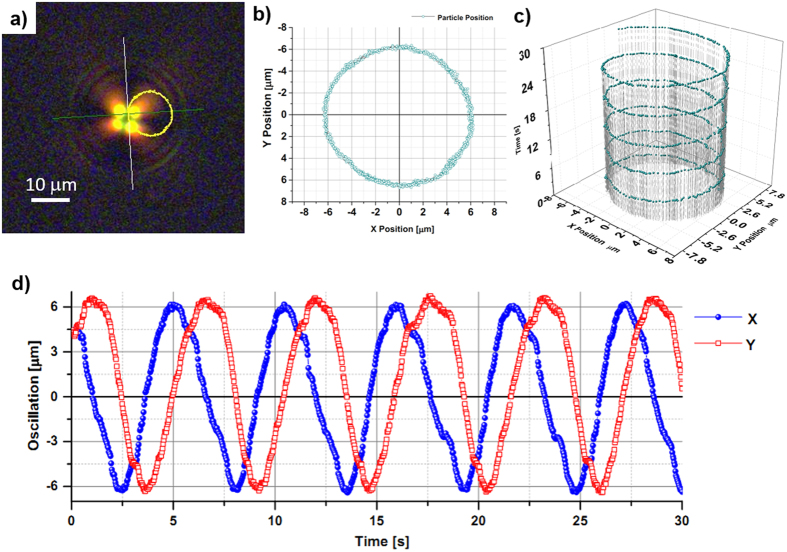
Orbital trajectory analysis for 1000 frames on 30 seconds of video 2. (**a**) Correspond to a capture from the supplementary video 2 after perform the trajectory analysis of the centroid of the particle, the calculated orbit is indicated by the yellow ring, (**b**) show the graphic of the orbit with respect to the beam axis on the captured images by the CCD camera, the calculated diameter of the orbit was approximately 13 μm (**c**) corresponds to the 3D graphic trajectory along the time of 30 *s* and (**d**) indicates the oscillation during the time for both coordinates *x* and *y*.

**Figure 6:**
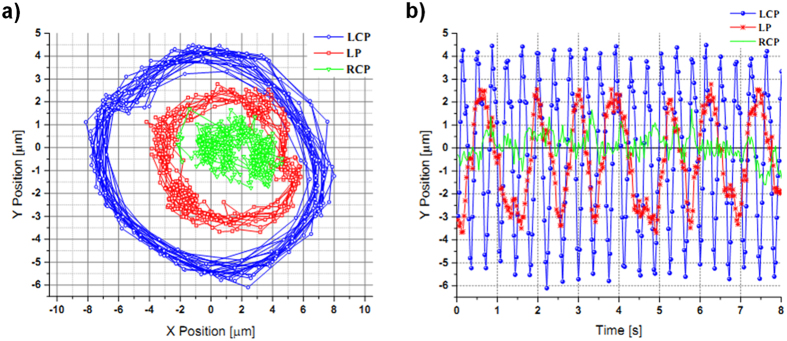
Analysis of orbital trajectories for each polarization state. On the graphic are plotted the experimental data obtained when the polarization state passes from LCP to LP and then to RCP. The data are obtained from the supplementary video 3. In (**a**) are reported trajectories of the particle and in (**b**) the projection of the position along y-axis.

For a wide particle size distribution, we observe orbital rotation around the centre of the beam. In Figs 5 and 6 we report the trajectory analyses of the orbital motion observed for particles with radius 10 μm and 15 μm, respectively (see supplementary videos 2 and 3).

As shown in Fig. 5, the particle describes a circular trajectory around the beam axis with a diameter of about 13 μm and a rotational frequency of 0.2 Hz.

In Fig. 6 we report the trajectories of a particle with *a* = 15 μm by changing the polarization of the trapping beam from LCP to LP and then to RCP. We observe that the orbital rotation of the chiral particle around the centre of the beam axis for LCP and LP light maintains always the same sense of rotation (clockwise) (see supplementary video 3). The diameter of the orbit and the rotational frequency reduce from 15 μm and 2 Hz, for LCP, to 10 μm and 0.6 Hz, for LP trap, respectively. However, for RCP light no rotation was observed and the particle is firmly trapped on the beam axis, see Fig. 6.

**Figure 7:**
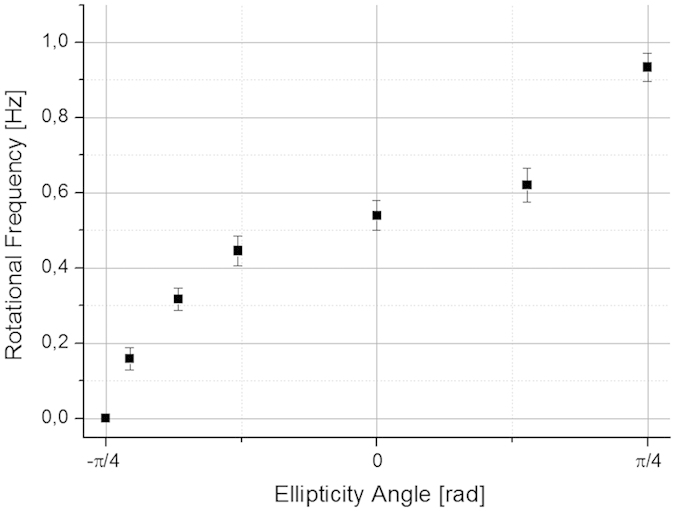
Rotational frequency of particles versus the ellipticity angle of the trapping light. On the graphic is plotted the rotational frequency of a particle with a radius *a* ≅ 3 μm spinning on the beam axis as a function of the ellipticity angle φ of the light.

In Fig. 7 we show the rotational frequencies measured for a particle spinning on the Gaussian beam axis (*a* ≅ 3 μm) as a function of the ellipticity angle φ of the light.

### Optical torque of unpolarized light on chiral particles

UP light, i.e. light with any polarization structure, is usually characterized by taking into account some properties: the invariance with respect to rotation of an orthogonal basis around the propagation axis, the symmetry with respect to an interchange of left and right-handed circular polarization and the invariance with respect to the phase retardation^39^. Ordinary light sources such as incandescent and fluorescent bulbs, LED spotlights, fiber optic illuminators and sunlight have structure of UP light. Depending on the above features, UP light can be also regarded as a racemic entity, whose random phases of the fields should not affect the optomechanical phenomena, related to the reflected or transmitted light power. Such concept is physically more understandable by considering a quantum mechanical description, where the transfer of angular momentum is discussed in term of the helicity and the spin of the photons, instead of the light polarization.

**Figure 8:**
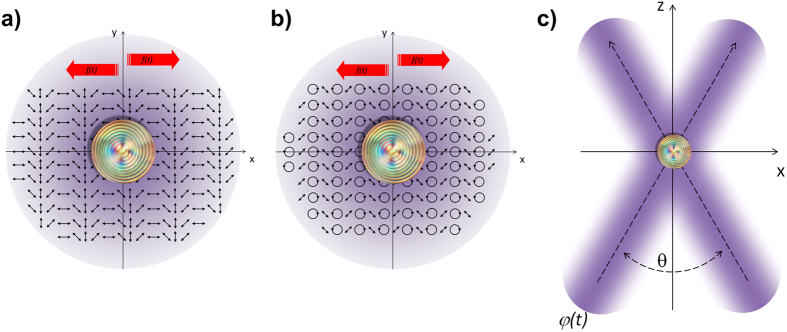
Unpolarized light field. (**a**) *x-y* uniform intensity profile within the superposition region of two opposite circularly polarized beam and polarization spatial distribution. The polarization pattern has a spatial periodicity smaller than the particle size, in experiment the value of Λ is about 5 μm (see Methods), ensuring the condition of all linear polarization states impinging on the particle; (**b**) *x-y* uniform intensity profile within the superposition region of two orthogonal linearly polarized beams and polarization spatial distribution; (**c**) *x-z* plane showing the superposition area of the beams with a 20 μm spot size, the longitudinal region of superposition is larger than the particle size, in the present case about 80 μm.

To obtain a UP light, avoiding light diffusion and preserving Bragg reflection, we exploit the interference of orthogonally polarized Gaussian laser beams^40^,^41^,^42^,^43^ (see Fig. 8): we tested the interference of two linear polarized *s* and *p* beams, as well as two opposite circularly polarized (RCP and LCP) beams of equal intensity. In such interference light fields, no intensity modulation is expected in the interference region, but only polarization patterns as the ones reported in Fig. 8a, whose longitudinal extension depends on the crossing angle between the beams. A random phase shift *ψ*(*t*) between the interfering beams (Fig. 8b) has been introduced to move the polarization patterns, obtaining a light field whose polarization state varies both temporally and spatially.

In the case of orthogonal *s* and *p* polarizations, the resulting optical electric field and intensity profile are respectively:


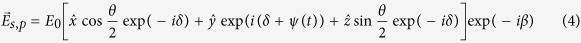



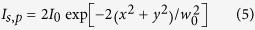


where *n*_*m*_ is the refractive index of the medium, 
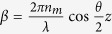
, 
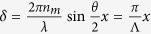
 and 
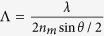
 is the spatial periodicity of the pattern.

In the case of opposite circular polarizations, the resulting optical electric field and intensity profile are respectively:


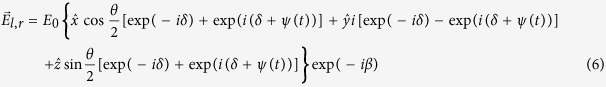



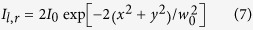


for small crossing angle *θ*.

The details of the procedure and experimental setup are described in the Methods, for both configurations.

A spatial periodicity of 5 μm has been set to ensure the above condition for Bragg particles (at 488 nm) to which corresponds a longitudinal extension of the superposition region of about 80 μm, larger than the trapped particle (about 15 μm diameter).

Clockwise rotation of trapped particles are observed both in orthogonal *s*-*p* configuration (supplementary video 4) and in opposite circular configuration (supplementary video 5), demonstrating that also exploiting UP light all optical manipulation tasks, i.e. trapping and displacement as well as rotation of the particle, can be achieved.

## Discussion

We demonstrate that spin and orbital motion of chiral microparticles can be performed with ordinary achiral light, such as LP or UP light, by simply tuning the light wavelength. The spinning of the particle induced by LP and UP light is a clear evidence of the racemic resolution of the achiral light enabling transfer of the AM. However the observed orbital motion cannot be accounted for by the transfer of OAM from light to matter, since it is absent in Gaussian beams, but it can be explained taking into account the combined effect of linear and angular momentum exchange when the trapping position is expected on a circular orbit around the beam axis. In such case, the particle is located away from the beam axis and is asymmetrically illuminated by the Gaussian beam, thus a deviation of the spinning angular velocity from the beam axis occurs due to a transverse component of the light AM^38^, as well as an additional torque perpendicular to it could be responsible of the orbital motion of the particles in the transversal plane which sense depends on their spinning. Deep calculation of the optical torque when such asymmetric spherical particle trapping by Gaussian beam occurs needs to be performed.

It worth noting that trapping and rotation could be achieved by using LP or UP broadband or even white light, providing that the portion of the spectrum out of the materials stop band carries enough linear momentum to trap the particles and the portion within the stop band transfer angular momentum upon selective reflection.

In conclusion, we demonstrate the chiral resolution of LP and UP light through transfer of SAM exploiting the interaction with chiral objects. Based on the fact that LP or UP light can be considered as racemic entities, i.e. composition of equal left- and right-handed components, asymmetric interaction of their components with such media results in a net SAM transfer. The interplay between linear and angular momentum transfer enables to control the object position and rotation. The experimental demonstration has been performed controlling the reflectivity of chiral Bragg particles, which exhibit circular selective reflection, by means of the light wavelength and the stop-band spectrum. With the same aim, circular dichroism can be also used to obtain the same effects^31^,^32^. In such cases new opportunities for optomechanics can be foreseen by fostering material science achievements, such as nanostructured chiral materials which can broaden this operation principle at the nanoscale. The helicoidal 3D nanostructures with circular dichroism in the visible range^44^ are a concrete example. The reported results open new routes for optomechanical technologies based on engineering materials instead of shaping the light, where simple white light sources coupled with wavelength selective elements can be implemented in optomechanic tools or micromachines to easily perform any optically controlled mechanical task.

## Methods

### Chiral particles synthesis

The left-handed chiral microparticles are prepared through the following steps. First, a pure reactive mesogen contained in RMS03-001C blend (Merck, Germany) is obtained extracting its solvent PGMEA (propylene glycol monomethyl ether acetate) by vacuum evaporation at 90 °C. Thus, this is mixed with a left-chiral dopant (ZLI-811, Merck) at concentration of 23% wt. yielding a CLC phase. Then, a CLC droplet emulsion is prepared with type I ultrapure water (18.2 MΩ cm resistivity at 25 °C, < 5 ppb total organic carbon). The values of the extraordinary and ordinary refractive indices of the reactive mesogen at 500 nm are respectively n_//_ = 1.68 and n_⊥_ = 1.53. The emulsion is polymerized in nitrogen environment for at least 4 hours under a 2 *mW cm*^−2^ intensity UV lamp (Mega Electronics lamp mod. LV202-E) having emission wavelength centred at 350 nm. The size of the solid chiral particles produced by this method ranges from hundreds of nanometres up to tens of microns. The resulting helicoidal pitch *p*, is approximately 0.3 μm. Finally, a few tens of microliters of the chiral particles suspension are loaded in a small glass chamber for OT experiments.

### Optical setup

The experimental proof to demonstrate chiral resolution of SAM from LP light is performed with an linearly polarized Argon-Ion laser beam at wavelength λ = 488 nm, directed by a dichroic mirror (DM) towards a 60X microscope objective (NA 0.85) and with a power on the sample plane of 100 *mW*. The beam is focused onto a sample chamber containing the chiral particles dispersed in water. A quarter-wave plate, placed before the microscope objective is used to control the light polarization state. To observe the radial structure of the particles, we use a crossed polarizer and analyser system with the sample in between. The sample was illuminated with a white light fibre and imaged onto a charge-coupled device (CCD) camera DCU2233 (Thorlabs).

### Video analysis

Videos are obtained with a CCD camera DCU2233 (Thorlabs), progressive scan with resolution 1024 × 768 pixel, each square pixel size is 4.65 μm. The analysed videos are split on image frames for each 0.03 s, two-dimensional centre-of-symmetry tracking is performed on each image to find the centres of each tracking sphere in order to calculate the trajectories with the software Able Particle Tracker.

### Unpolarized light set-up

**Figure 9:**
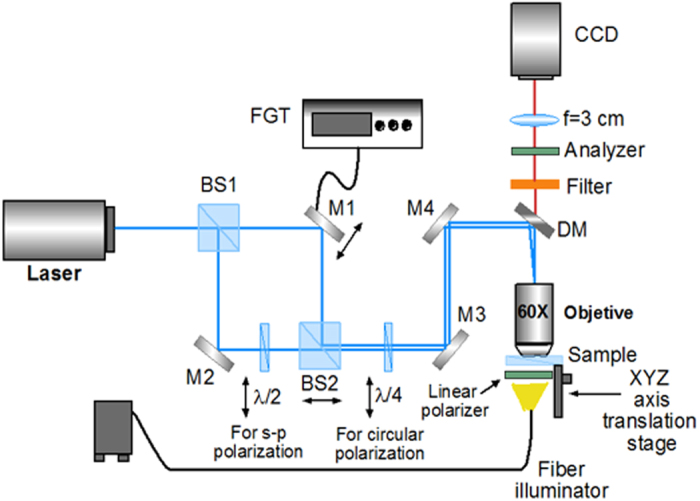
Experimental set-up for UPL optomechanical experiment. A *s*-polarized Argon ion laser beam at λ = 488 nm, is sent into a Mach-Zehnder interferometer. The half-wave plate (λ/2) in one of the two arms converts into *p* the polarization of the beam propagating in it. The two s- and p- polarized beams are directed towards the 60x microscope objective by a dichroic mirror (DM), and interfere on the sample. A quarter-wave plate (λ/4) is used to switch to right and left circular the interfering beams. BS1 and BS2 are beam splitters. M1, M2, M3, M4 are mirrors. A computer controlled CCD camera and a fiber illuminator have been used to image the sample.

The experimental set-up is reported in Fig. 9. A *s*-polarized Argon-ion laser beam at λ = 488 nm is sent into a Mach-Zehnder interferometer. The quarter-wave plate (λ/4) at the exit of the interferometer changes the polarization state of the light beams from linear to circular (left or right, respectively). Mirrors labelled as M2, M3, and M4 are fixed. The two beams are directed towards a 60X microscope objective through a dichroic mirror (DM), focusing the resulting polarization pattern on the sample. A crossing angle of few degrees was set in order to have a spatial periodicity of the polarization pattern Λ of about 5 μm. The spatial periodicity of this pattern has been tuned by shifting the beam splitter BS2. The phase variation of one of the two interfering beams was performed in order to move the polarization fringes, by moving the mirror M1 along the surface normal. The mirror is mounted on a 3 axis-piezoelectric system, which is driven by an arbitrary function generator (MTD693A, Thorlabs). A stochastic function f(t) was set in order to have a random phase *ψ*(*t*) of the light beam at the exit of one arm of the interferometer. The sample position is adjusted via a three-axis translation stage. The (CCD) camera and a white light in fiber have been used to image the sample placed between polarizer and analyser in order to visualize the characteristic cross pattern of the spherical chiral particles.

## Additional Information

**How to cite this article**: Hernández, R. J. *et al*. Chiral resolution of spin angular momentum in linearly polarized and unpolarized light. *Sci. Rep*. **5**, 16926; doi: 10.1038/srep16926 (2015).

## Supplementary Material

Supplementary Information

Supplementary Video S1

Supplementary Video S2

Supplementary Video S3

Supplementary Video S4

Supplementary Video S5
