# Supplementary material for: Chiral resolution of spin angular momentum in linearly polarized and unpolarized light
Source: Sci Rep. 2015 Nov 20;5:16926. doi: 10.1038/srep16926 (PMC4653716; doi:10.1038/srep16926)
Supplement: Supplementary Information [file srep16926-s1.doc]

**Supplementary information**

**Chiral resolution of spin angular momentum in linearly polarized and unpolarized light**

R.J. Hernández^2^, A. Mazzulla^2^, C. Provenzano^1^, P. Pagliusi^1,2^, and G. Cipparrone^1,2*^

*^1^Physics Department, University of Calabria, Ponte P. Bucci, Cubo 33B, 87036 Rende (CS), Italy*

*^2^CNR-Nanotec, LiCryL lab., Ponte P. Bucci, Cubo 33B, 87036 Rende (CS), Italy*

^*^E-mail: [gabriella.cipparrone@fis.unical.it](mailto:gabriella.cipparrone@fis.unical.it),

In the ray optics approach, the equation to obtain the transversal gradient force exerted by the light beam onto the spherical particle of radius *a*, in a plane *z=const*, can be written as [1]:

$F_{g}=\frac{n_{m}a^{2}}{c}\int_{0}^{\frac{\pi}{2}} \int_{0}^{2\pi} I(\rho,\phi,z)\cos\theta\left[R\sin(2\theta)-T^{2}\frac{\sin(2\theta-2\eta)+R\sin(2\theta)}{1+R^{2}+2R\cos(2\eta)}\right]\cos\varphi\sin\theta d\varphi d\theta,$ (1)

where, T and R are the transmittance and the reflectance, respectively, derived from the Fresnel coefficients, *n_m_* is the refractive index of the host medium (water), for an incident ray the incidence angle is *θ* and *η* is the refraction angle. In addition,$I(\rho,\phi,z)=I_{0}\exp\left[-\frac{2\rho^{2}}{\omega^{2}}\right]$ is the intensity distribution of the Gaussian beam given as function of the distance ρ from the axis beam to the sphere centre and their radius *a*. The integration is performed over the illuminated hemisphere of the microsphere. Therefore, it should be stated the position of the particle with respect to the intensity's beam distribution considering two coordinated systems indicated on the figure.


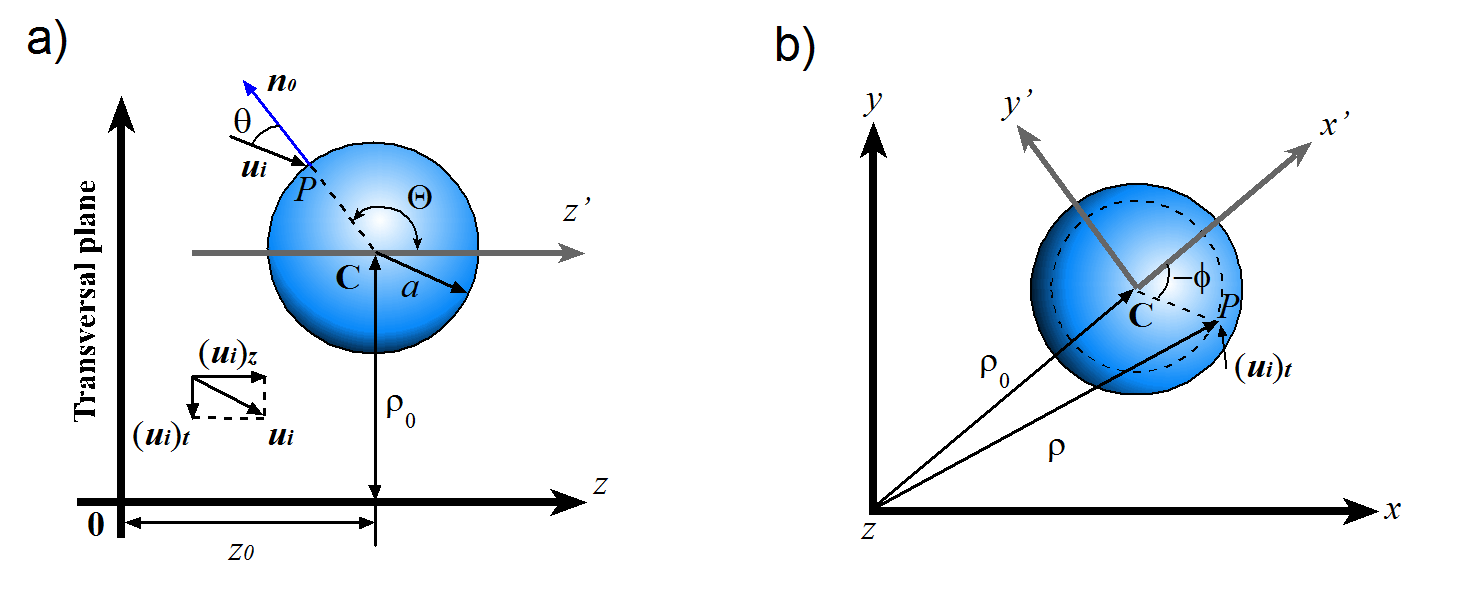


**Figure S1:** Geometrical parameters for ray model. Model with rays for a dielectric sphere under the influence of a light beam propagating along the z direction. a) Side view, b) front view.

On the reference frame *S* on part a) of the figure, the spherical particle of radius *a,* is located on the point *C* of the coordinate system associated to the light beam. On this coordinate system, the origin corresponds to the symmetry centre of the transversal intensity distribution in the beam, which on cylindrical coordinates is given in terms of (*ρ, ϕ, z*). Is also represented the incidence point *P* of the ray at incidence angle θ with respect to the normal surface and the distance ρ_o_ between the *z* axis and the centre of the sphere. For radial particles, θ is also the angle between the incident ray and the axis of the helical supramolecular structures. Due to symmetry considerations, we can look only at the gradient force along the *x* direction without loss of generality.

The second reference frame *S´* is located on the centre of the sphere and is not a fixed frame system because it moves along with the sphere. On the figure, are represented the angles of the sphere where *φ* is the polar angle and *θ* is the azimuthal angle. The parameter *ρ* is the distance between the axis and the incidence point *P*. Finally, *z_o_* is the distance between the reference plane *z=0* and the centre of the sphere. Then, both reference frame systems are related by:

$$\begin{matrix} \rho=\left[\rho_{0}^{2}+a^{2}\sin^{2}\theta+2\rho_{0}a\sin\theta\cos\varphi\right]^{1/2}, \\ z=z_{0}+a\cos\theta, \end{matrix}$$

On the other hand, the expression for the components of the scattering force along the propagation direction axis *z*, can be evaluated from:

$F_{s}=\frac{n_{m}a^{2}}{c}\int_{0}^{\frac{\pi}{2}} \int_{0}^{2\pi} I(\rho,\phi,z)\cos\theta\left[1+R\cos(2\theta)-T^{2}\frac{\cos(2\theta-2\eta)+R\cos(2\theta)}{1+R^{2}+2R\cos(2\eta)}\right]\sin\theta d\varphi d\theta$ (2)

The expressions of intensity and the forces are given in function of *ρ*, to change back to the Cartesian coordinate system, the additional equations relating both coordinate systems are given by:

$x=x_{0}+a\cos\varphi\sin\theta$,

$y=y_{0}+a\sin\varphi\sin\theta$,

$z=z_{0}+a\cos\theta$,

where *x*_o_ and *y*_o_ are the parameters that indicate the displacement of the particle centre with respect to the propagation axis.

[1] R. Gussgard, I. Brevik, and T. Lindmo, "Calculation of the trapping force in a strongly focused laser beam," J. Opt. Soc. Am. B **9**, 1922-1930 (1992)
